# Low Titer *Pneumocystis jirovecii* Infections: More than Just Colonization?

**DOI:** 10.3390/jof2020016

**Published:** 2016-05-28

**Authors:** Alexander Prickartz, Jessica Lüsebrink, Soumaya Khalfaoui, Oliver Schildgen, Verena Schildgen, Wolfram Windisch, Michael Brockmann

**Affiliations:** 1 Lungenklinik Merheim, Kliniken der Stadt Köln gGmbH, Universität Witten-Herdecke, Alfred-Herrhausen-Straße 50, Witten 58448, Germany; Alexander.prickartz@malteser.org (A.P.); windischw@kliniken-koeln.de (W.W.); 2 Institut für Pathologie, Kliniken der Stadt Köln gGmbH, Klinikum der Privaten Universität Witten/Herdecke mit Sitz in Köln, Ostmerheimer Str. 200, Köln/Cologne D-51109, Germany; luesebrinkj@kliniken-koeln.de (J.L.); khalfaouis@kliniken-koeln.de (S.K.); schildgeno@kliniken-koeln.de (O.S.); schildgenv@kliniken-koeln.de (V.S.)

**Keywords:** PCP, *Pneumocystis jirovecii*, pneumonia, colonization, chronic cough, successful recovery, chronic infection

## Abstract

Non-pneumonia *Pneumocystis jirovecii* colonization is thought to occur frequently in immunocompetent individuals. The aim was to analyze if *P. jirovecii* low-titer detections have more impact than just colonization. From our total cohort of patients for which *P. jirovecii* testing by qPCR was requested, we selected exclusively those that were fully immunocompetent. Patients were defined as fully immunocompetent if they did not receive immunosuppressive therapy, displayed regular antibody titers, and did not suffer from acquired, inherited or autoimmune diseases. Only those patients with complete medical records available were included. A retrospective analysis identified patients with *P. jirovecii* colonization and successful antibiotic therapy in response to laboratory pathogen detection. We identified 30 fully immunocompetent patients with *P. jirovecii* colonization suspected to suffer from infection with the pathogen, but with milder symptoms than pneumonia. All patients were successfully treated with cotrimoxazole against *P. jirovecii* and resolved from chronic cough and recurrent pulmonary infections. The fact that all patients displayed recovery from their clinical symptoms gives raise to the hypothesis that *P. jirovecii* infections may also occur in immunocompetent patients but with milder symptoms.

## 1. Introduction

Respiratory infections remain a major cause of death in children and elderly adults worldwide. Thereby, a leading cause of death in the weakest patients, children suffering from AIDS in non-industrialized countries, is infection with *Pneumocystis jirovecii*.

Organisms recognized as *Pneumocystis* have been found in many mammalian species investigated so far. They constitute a family of fungi showing a strict host-species specificity [1,2,3]. The species infecting specifically humans is named *Pneumocystis jirovecii*, and it can cause severe pneumonia in immunocompromised individuals (*Pneumocystis jirovecii* pneumonia, PCP), which may be recurrent and sometimes fatal. Despite a decrease in incidence due to the advent of the highly active antiretroviral tri-therapy, PCP remains the most common AIDS-defining infection [1,2,3], and is also of clinical importance in HIV-negative patients such as transplant recipients and those receiving chemotherapy for malignant diseases. PCP is in fact the second most frequent life-threatening invasive fungal infection worldwide with an estimated number over 400,000 per year [4].

*P. jirovecii* was identified 105 years ago [5,6,7] and, during this time span of more than a century, turned out to be a serious pathogen in airway infections. Major attention was drawn to this pathogen with the first wave of AIDS patients who suffered from *P. jirovecii* infections and died from PCP [8].

Hitherto *in vitro* culture of this pathogen has been unsuccessful, thereby making it difficult to assess possible therapeutic choices. In order to develop effective therapies against a pathogen such as *P.* *jirovecii*, this pathogen needs to be efficiently cultured to be studied. Despite generations of microbiologists that tried to establish an efficient culturing system for *P. jirovecii*, all previous attempts to establish such a system failed. This serious gap could be closed by a novel and potent cell culture system that enable growths and passaging of *P. jirovecii*. Using an approach that is novel for the field of microbiology, we succeeded in solving this issue. We used novel air-liquid interface cultures that simulate the natural locus of *P. jirovecii* infection, *i.e.*, the airway epithelia. By using this approach the colleagues were able to grow *P. jirovecii*, and the number of fungus particles significantly increased [9].

There is a high rate of *P. jirovecii* detections in the bronchoalveolar fluid of patients without typical signs of pneumonia. Low titers of *P. jirovecii* are believed to lead to asymptomatic pulmonary colonization with *Pneumocystis jirovecii* in adult patients with HIV infection, rather than to true infection [10,11,12,13,14,15,16]. So far it was assumed that patients of this cohort will solely suffer from an infection with *P. jirovecii* when being immunosuppressed. Further, there are many HIV-negative patients in whom *P. jirovecii* DNA was detected at low copy numbers which was believed to reflect the background noise from the high rate of seroprevalence; however, this concept has not been proven and requires additional investigations.

Based on our observations in cell culture, in which we observed serious cytopathic effects that clearly led to damage of the tissue, we doubted that these colonizations were harmless but hypothesized that the detection of *P. jirovecii* in clinical samples is meaningful for the patients’ well-being and health. Consequently, the aim of this study was to elucidate the clinical features of low-titer, subtle *P. jirovecii* infections in patients without *Pneumocystis* pneumonia (PCP). Therefore, we made use of a previously published sensitive qPCR in order to identify *P. Jirovecii*-positive immunocompetent patients.

## 2. Materials and Methods

The study was performed as a retrospective analysis. Between October 2012 and April 2014, 533 bronchoalveolar lavages (BAL) were sent to our institution for qPCR testing for *P. jirovecii*. BAL samples were tested for *P. jirovecii* by qPCR as described previously [9,17]. Of the samples, 129 were positive for *P. jirovecii*. From this cohort of *P. jirovecii*–positive patients, we extracted exclusively the HIV-negative patients with complete medical records regarding BAL analyses, background history, and antibiotic treatment using the clinical patient information system. Patients were defined as fully immunocompetent if they did not receive immunosuppresive therapy, displayed regular antibody titers, and did not suffer from acquired, inherited or autoimmune diseases. Thirty patients fulfilled the criteria mentioned before and were included in this study. None of these patients showed clinical and radiological signs of *Pneumocystis* pneumonia. In this cases, initial *P. jirovecii* testing was requested from lack of alternative diagnoses. In contrast, PCP was defined by increased both-sided opacification in the lower lung, widespread pulmonary infiltrates, shortness of breath, reduced PaO_2_, and positive PCR and/or microscopy.

The study was performed in accordance with an ethical vote from the local ethical committee (No. 75/2013). All procedures were in accordance with the Declaration of Helsinki and were performed solely for diagnostic purposes; thus, no additional processes were performed for study purposes.

Laboratory investigations to detect further facultative or obligate respiratory pathogens (including viruses, fungi, and bacteria) were conducted using Respifinder Smart22, Meningofinder Custom Assay (both Pathofinder, Maastricht, The Netherlands), *P. jirovecii* PCR, and conventional microbiological screening methods [9].

For statistical analyses a two-tailed Student’s *t*-test was performed.

## 3. Results

In principle, *P. jirovecii* testing is performed in at risk and immunocompromised patients suffering from respiratory infections with an increased risk of developing PCP. Together with our pneumology clinic, which includes intensive care units with the possibility of ECMO (extracorporeal membrane oxygenation), we have established a diagnostic algorithm that is based on a multiplex assay for the most common respiratory pathogens and that, in the case of negative results, is complemented by *Aspergillus galactomannan* ELISA testing and *P. jirovecii* qPCR. This algorithm is used in the case of patients with symptoms of respiratory infections, *i.e.*, to avoid pneumonia by early diagnosis. For this reason we also test otherwise healthy patients for *P. jirovecii*. During the study period, the laboratory received 976 BAL samples in total. On request, 533 BALs (54.6%) were tested for *P. jirovecii*; of those, a PCR-positive test result was obtained in 129 BALs (13.2%). No other pathogens were detected. In detail, the BALs were negative for influenza viruses, parainfluenzaviruses 1–4, RSV (Respiratory Syncytial Virus), HMPV (Human Metapneumovirus), coronaviruses NL63, OC43, 229E, and HKU-1, adenoviruses, *Mycoplasma pneumoniae*, *Mycobacteria*, mumps, measles, human herpesviruses 1–8, parechoviruses, rhinoviruses and enteroviruses*, Legionella pneumoniae*, *Chlamydia pneumoniae*, *Aspergillus* and *Bordetella pertussis* by molecularbiological assays and culturing.

In the cohort of *P. jirovecii*-positive patients, 75 were male (58%, median age 62 years, mean age 58.63 years) and 54 were female (42%, median age 62.5 years, mean age 60.89 years). In 38 (29%) patients a lymphocytosis (≥10% lymphocytes) was observed, and 71 (55%) patients showed a neutrophilia (≥10% neutrophilic granulocytes).

Within a time span of four years, we retrospectively identified 30 immunocompetent patients with qPCR-confirmed *P. jirovecii* infection that received antibiotic treatment active against this fungus, and in which no further respiratory pathogens were identified. The overview on these 30 immunocompetent patients is summarized in Table 1.

The patients suffered from chronic cough infections. The average age was 58 years (22–77), 30% were female (*n* = 9). Eight patients were active smokers, nine were former smokers and 12 were never smokers; for one patient this information is missing. The average *P. jirovecii* copy number was 1.51 × 10^8^ (2.2 × 10^2^–2.11 × 10^9^) copies per ml BAL of the mtLSU gene. LDH (lactate-dehydrogenase) was elevated in 50% of patients (*n* = 15), with an average of 297 U/L (158–672, reference <250 U/L). The Pearson coefficient was 0.231 and *r*^2^ was 0.0534, both indicating a positive statistical correlation.

**Figure 1:**
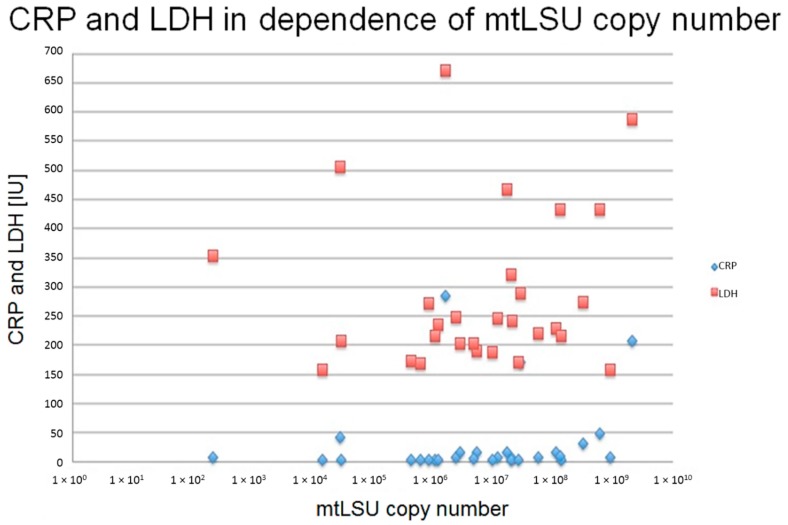
The figure shows the correlation between LDH, CRP, and the copy number of mtLSU from BAL fluids. LDH is elevated with dependence of the copy number, *i.e.*, the more mtLSU copies, the higher the LDH concentration. This effect was not present for CRP. The Pearson coefficient was 0.231, *r*^2^ was 0.0534, both indicating a positive statistical correlation. However, the correlation is statistically significant only for CRP but not for LDH.

CRP (C-reactive protein) was elevated in 53% (*n* = 16), with an average of 44.8 mg/L (<3–285, reference <5 mg/L), and correlated with higher mtLSU copy numbers (Figure 1).

Thereby we observed both the positive Pearson’s coefficient and the Pearson’s coefficient of determination (Pearson: 0.424; *r*^2^: 0.18). As both coefficients are positive, this indicates a positive correlation. Keeping in mind that both coefficients solely allow us to discriminate between positive or negative correlations but not with the quality of an existing correlation, it could be assumed that at least a (weak) positive correlation exists between a high *P. jirovecii* copy number in the PCR and the elevation of the CRP levels. Both correlations tested for statistical significance using the SPSS 22 software using the two-sided correlation and significance test. The significance level of the correlation between the copy number and LDH was *p* = 0.480, the significance level (two sides) of the correlation between the CRP and copy number was *p* = 0.017; thus, both the correlation between the CRP and copy number was statistically significant at a 95% confidence interval, while the correlation to LDH was not statistically significant. However, no further correlations were observed between the levels of *P. jirovecii* copies, PaO_2_ < 75 mmHg, LDH, and CRP. It is worth noting that one patient without pneumonia had an mtLSU copy number >10^9^ copies per ml BAL fluid.

In 53% of patients (*n* = 16) the pO2 was decreased, but in only 13% (*n* = 4) it was below 55 mmHg. Classical radiological signs of *P. jirovecii* infections were observed in four patients (although without pneumonia), thus most likely presenting a precursor of pneumonia or an intermediate course of the infection. All patients recovered during antibiotic therapy with Cotrim^®^, which is the standard therapy in our hospital, and got rid of the symptoms and cleared the fungus. Patients received 1 × 960 mg Cotrim three times per week for 21 days (recommended dose for prophylaxis).

## 4. Discussion

The detection of *P. jirovecii* in the BAL is an alarming diagnosis as this pathogen is capable of inducing a life-threatening clinical condition, *Pneumocystis pneumonia* (PCP). PCP was frequently observed in malnourished children after World War II and in AIDS patients during the first two decades of the HIV pandemic [8,18,19,20,21,22]; both times, the affected patients suffering from PCP were not fully immunocompetent due to the HIV infection or malnutrition. Due to better nutrition conditions (at least in industrialized countries) and optimized HIV therapies preventing patients from the status of AIDS, the number of lethal PCPs decreased in the past and the fungus was lost from the focus of research, although it still remains a major health problem. Novel therapies, e.g., against cancer, allergies, rheumatoid diseases, *etc.*, as well as organ transplants are combined with immunosuppression, giving rise to new, serious infections with *Pneumocystis* [23,24,25,26,27,28].

Furthermore, it is a well-known fact that there is a high seroprevalence against *P. jirovecii* and it is discussed that at least 50% of the entire population are carriers of *P. jirovecii* particles [29,30], although the majority of these carriers will never experience an exacerbation in the form of PCP or similar conditions.

In the present pilot study, we identified 30 fully immunocompetent patients that, according to clinical definitions, would have been classified as solely colonized with *P. jirovecii* rather than being infected [10,11,12,13,14,15,16]. None of these 30 patients with a full documented medical history suffered from clinical or radiological signs of PCP, but all reported recurrent airway infections and chronic cough. Being negatively tested for all important respiratory viruses and bacteria, all patients tested positive for *P. jirovecii* by qPCR. The titers were approximately 10^8^ mtLSU copies per ml of BAL fluid, which corresponds to a 10^4^–10^6^ lower number of nuclear genomes (*i.e.*, a 10^5^–10^7^ lower number of fungal particles) [9]. Although there is no universal guideline, there is consent that higher *P. jirovecii* titers are more frequently associated with pneumonia, while lower titers often indicate a milder colonization; however, there is no consent on the cut-off values, nor is there any standardization or consent that clearly discriminates between colonization and infection [10,11,12,13,14,15,16]. A novel approach was made by Louis *et al.*, who, for the first time, tried to harmonize the cut-off value, while also addressing the general problem that international standard cut-off values are still missing due to the different targets of qPCR (e.g., mtLSU gene *vs.* MSG (multicopy surface glycoprotein gene)) [31].

Our study clearly shows that patients with these putative colonizations in fact suffered from chronic cough likely associated with *P. jirovecii* infections milder than PCP, but which were clinically relevant for the life quality of the individual patients. All of the 30 immunocompetent patients included in this study fully recovered after the treatment with antibiotics active against *P. jirovecii*; thus, the patients were more than solely colonized but experienced a serious infection. It also became obvious from our study that despite the fear of PCP in immunosuppressed patients, *Pneumocystis* is an underestimated pathogen in the immunocompetent cohort suffering from chronic airway diseases.

Although at the present stage it remains unclear how many chronic infections with *P. jirovecii* occur, the results of our study suggest that the fungus might also act as a relevant pathogen in patients with low titers and is more than an innocent bystander in immunocompetent patients. Further studies are, however, required to elucidate the frequency of low-titer infections and the percentage of associations with chronic airway diseases. In concert with novel diagnostic tools to isolate the fungus [9], this will contribute to optimized therapy and prevention concepts.

A bias in our study is that we were not able to include all 129 PCR-positive patients in the study. The optimal case would have been to analyze all 129 PCR positive patients, but full clinical data sets were available for only 30 patients that were included in this study. Moreover, we could not exclude the possibility that the 30 cases which we reported suffered from putatively self-limiting infections and thus did not require the treatment, in spite of the fact that the treatment obviously was successful and eliminated the clinical symptoms. In future studies it will be necessary to analyze how many detected *P. jirovecii* cases with low fungal titers are associated with chronic respiratory symptoms. Moreover, the frequency of *P. jirovecii* colonization needs to be elucidated, as thus far mainly hospitalized patients will be tested for *P. jirovecii*, but the number of colonized patients in the non-hospitalized cohort may differ. In any case, it should be recommended that in addition to fluorescence-based and classical microscopy, molecular testing should be routinely performed for suspected *P. jirovecii* infections and colonization, as the classical methods would have missed virtually 100% of our successfully treated patients.

qPCR is a powerful tool to detect occult *P. jirovecii* infections that, according to previous assumptions, were classified as colonizations rather than infections. Surprisingly, the chronic coughs in our patient cohort were resolved by antibiotic therapy that was administered in response to the *P. jirovecii*–positive PCR result. Thus, foremost attention should be drawn to *P. jirovecii* PCR in patients testing negatively for other respiratory pathogens, even if they do not suffer from clinical or radiological signs of PCP.

## Figures and Tables

**Table 1:** Overview on the patient characteristics of 30 fully immunocompetent patients positive exclusively for *P. jirovecii* extracted out of the entire cohort for which full medical records were available.

Parameter	(*n* = 30)
Female (*n* (%))	9 (30%)
age (years)	58 (22–77)
Active smokers	8 (27%)
*P. jirovecii* mtLSU copies per ml BAL	mean: 1.51 × 10^8^ (range: 2.2 × 10^2^–2.11 × 10^9^)
PaO_2_ (mmHg)	mean: 67.9 (range: 49.5–91.9)
PaO_2_ < 75 mmHg	*n* =16 (53%)
PaO_2_ < 55 mmHg	*n* = 4 (13%)
LDH (U/L) (Ref. < 250)	mean: 297 (range: 158–672)
LDH elevated	*n* = 15 (50%)
CRP (mg/L) (Ref. < 5)	mean: 44.8 (range: <3–285)
CRP elevated	*n* = 16 (53%)
BAL: elevated total cell count	*n* = 9 (30%)
BAL: Lymphocytary Alveolitis	*n* = 7 (23%)

## Abbreviations

The following abbreviations are used in this manuscript:

PCP	Pneumocystis pneumonia
qPCR	quantitative PCR
BAL	broncho-alveolar lavage
